# Novel Four-Way Variant Translocation, t(1;9;22;16)(q21;q34;q11.2;q24), in a Patient with Chronic Myeloid Leukemia

**DOI:** 10.3390/diagnostics14030303

**Published:** 2024-01-30

**Authors:** Han Joon Son, Jong Ho Lee

**Affiliations:** 1 Department of Laboratory Medicine, Yeungnam University Medical Center, Daegu 42415, Republic of Korea; hanjuni1224@naver.com; 2 Department of Laboratory Medicine, Yeungnam University College of Medicine, Daegu 42415, Republic of Korea

**Keywords:** chronic myeloid leukemia, four-way variant translocation, philadelphia chromosome, nilotinib

## Abstract

Chronic myeloid leukemia (CML) is characterized by the Philadelphia (Ph) chromosome resulting from the translocation of t(9;22)(q34;q11), producing the *BCR*::*ABL1* fusion gene. Variant Ph chromosome translocations, involving rearrangements in chromosomes other than 9 and 22, occur in 5–10% of CML cases. Herein, we report a unique case of a 36-year-old male with a four-way variant Ph chromosome. Conventional chromosomal analysis performed on bone marrow aspirate samples showed 46, XY, t(1;9;22;16)(q21;q34;q11.2;q24). Nested RT-PCR of the *BCR*::*ABL1* gene revealed a major *BCR*::*ABL* rearrangement. The treatment with nilotinib achieved a complete hematologic, cytogenetic, and molecular response after 12 months.

Chronic myeloid leukemia (CML) is a myeloproliferative neoplasm characterized by the Philadelphia (Ph) chromosome arising from the t(9;22)(q34;q11) translocation [1]. The oncogenic *BCR*::*ABL1* gene product, a fusion of the *ABL1* gene on chromosome 9q34 and the *BCR* gene on chromosome 22q11 yields an abnormal tyrosine kinase that dysregulates multiple signaling pathways involved in cell cycle control and apoptosis [2]. Variant Ph chromosomes have additional rearrangements beyond the classic translocation, observed in 5–10% of CML cases [3,4]. The prognosis for patients with variant Ph chromosomes has been variably reported, with some studies indicating poorer outcomes compared to those with the classic Ph chromosome, though others suggest no significant prognostic difference in the imatinib era [4,5,6,7,8]. Very rare are variant Ph chromosomes with complex four-way or five-way rearrangements. We present a novel case of a CML patient with a four-way variant Ph chromosome, t(1;9;22;16)(q21;q34;q11.2;q24).

A 36-year-old male with no significant medical history was evaluated by the hematology department in July 2022 after abnormal blood test results during a routine examination. He had lost approximately 20 kg over the past six months and had developed splenomegaly. His complete blood count (CBC) revealed an Hb level of 7.9 g/dL, a white blood cell (WBC) count of 195 K/μL, and a platelet (PLT) count of 653 K/μL. The differential count detailed 37% segmented neutrophils, 18% band neutrophils, 5% lymphocytes, 2% monocytes, 2% eosinophils, 2% basophils, 8% metamyelocytes, 18% myelocytes, 2% promyelocytes, 2% blasts, and an N-RBC count of 1/100 WBCs. Bone marrow examination revealed hypercellularity with a myeloid-to-erythroid ratio of 13.32:1 due to granulocytic proliferation. The increase in eosinophils, basophils, and monocytes was non-significant. The bone marrow cell differential count included 31.4% segmented neutrophils, 19.6% band neutrophils, 2.6% lymphocytes, 2.6% monocytes, 3.4% eosinophils, 2.0% basophils, 16.4% metamyelocytes, 13.4% myelocytes, 0.6% promyelocytes, 1.2% blasts, and 6.8% erythroid precursors. Conventional chromosomal analysis on bone marrow aspirate samples showed 20 cells with the karyotype 46, XY, t(1;9;22;16)(q21;q34;q11.2;q24) (Figure 1). Nested reverse transcriptase-PCR for the *BCR*::*ABL* gene indicated a positive major *BCR*::*ABL* rearrangement (b3a2, data not shown). A dual-fusion triple-color fluorescence in situ hybridization (FISH) analysis with whole blood samples detected a 1B1R1G1F pattern in 86.6% of cells (Figure 2). The patient was diagnosed with chronic phase CML with a variant Ph chromosome. Treatment began with hydroxyurea and nilotinib at 600 mg/day starting on the fifth hospital day. At the 12-month follow-up, CBC showed Hb at 14.7 g/dL, WBC at 8.03 K/μL, and PLT at 653 k/μL. No immature cells were noted in the peripheral blood smear. While the marrow aspirate was inadequate, the biopsy showed a normocellular marrow with normal maturation of myeloid and erythroid lineages. Quantitative PCR for *BCR*::*ABL* from marrow aspirate yielded an International Scale (IS) value of 0.01711, indicative of major molecular response (MMR). The patient achieved complete hematologic, cytogenetic, and molecular responses and is on nilotinib 600 mg/day as of October 2023.

Approximately 1000 cases of CML with variant Ph chromosomes have been reported to date. Among these, approximately 80 cases exhibit 4-way translocations, this case included [9]. Variant Ph chromosomes have been documented on various chromosomes, particularly at 1p36, 3p21, 5q13, 6p21, 9q22, 11q13, 12p13, 17p13, 17q21, 17q25, 19q13, 21q22, 22q12, and 22q13 [10]. The breakpoints at 1q21 and 16q24, as noted in the patient, are rare. A study encompassing 25 cases with variant Ph chromosomes highlighted chromosomes 1 and 16 as the most commonly implicated [7]. Furthermore, according to the Mitelman Database, the t(1;9;22;16)(q21;q34;q11.2;q24) translocation observed here represents the inaugural reported case of such a four-way translocation [9].

Two hypotheses have been advanced to elucidate the genesis of variant Ph chromosomes: a simultaneous, one-step mechanism involving three or more chromosomes and a sequential, two-step mechanism that follows the initial t(9;22) translocation. This latter multi-step process may signify clonal evolution and potentially portend an unfavorable prognosis [3,11].

**Figure 2:**
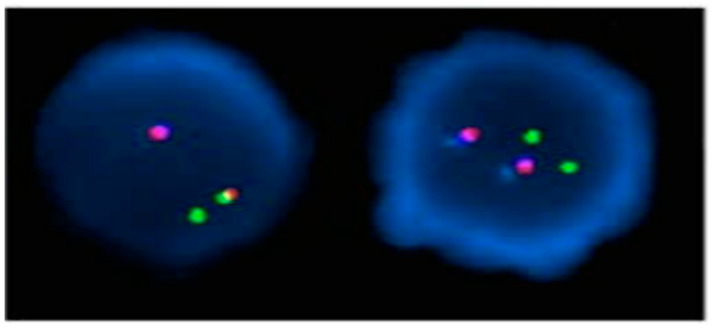
FISH analysis using the LSI *BCR*::*ABL* dual fusion triple color translocation probe. The method involves using DNA probes that are complementary to the *ABL1* gene on chromosome 9 and the *BCR* gene on chromosome 22. These genes are involved in the t(9;22)(q34;q11.2), and the probes hybridize to the nuclei of either interphase or metaphase cells. The Breakpoint Cluster Region (*BCR*) at 22q11.2 is identified by a green signal, while the *ABL1* oncogene at 9q34 is marked by a red signal, and the *ASS1* gene, also located at 9q34, is indicated by a blue signal. A cell negative for the t(9;22) typically displays a pattern of 2 blue, 2 red, and 2 green signals. However, in this study, out of 500 cells examined, 433 cells (86.6%) showed an unusual *BCR*::*ABL* rearrangement. The typical pattern for a 4-way variant translocation is 2 blue, 2 red, 2 green, and 1 fusion signals in 1-step mechanism and 2 blue, 1 red, 1 green, and 2 fusion signals in 2-step mechanism [4,11]. However, the atypical pattern observed in this study, characterized by 1 blue and 1 red signal on normal chromosome 9, 1 green signal on normal chromosome 22, and 1 fusion signal on der(22). It is apparent that deletions have occurred in both the *ABL1* and *BCR* genes.

Historical perspectives posited that variant Ph chromosomes could portend a graver outlook compared to the classical Ph chromosome, with deleting der(9) also linked to worse outcomes [6,12,13,14]. Conversely, contemporary research indicates that variant Ph chromosomes do not influence prognosis in the era of imatinib treatment, regardless of the number of chromosomes involved or deletion presence [4,11]. Nevertheless, specific cases, such as two patients with variants involving chromosome 5 but without tyrosine kinase domain mutations, have been associated with poorer prognoses, suggesting that specific chromosomes or breakpoints may indeed affect patient outcomes [7].

The patient under discussion achieved a major molecular response after 12 months of nilotinib treatment and has continued to progress favorably. The influence of variant Ph chromosomes on CML prognosis remains a topic of debate, and it is anticipated that this report of a rare four-way translocation will enrich the collective database, aiding in the refinement of our understanding of these chromosomal variations.

## Figures and Tables

**Figure 1:**
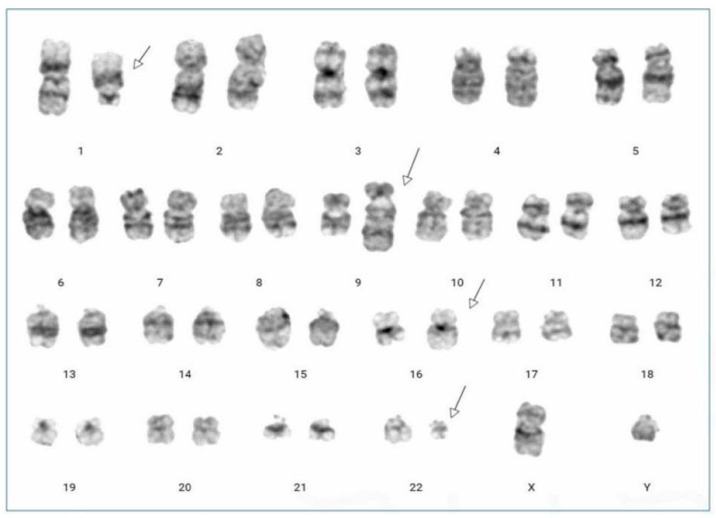
GTL-banding karyotypes of cultured bone marrow cells at a resolution of 400. All 20 observed dividing cells exhibit the t(1;9;22;16)(q21;q34;q11.2;q24). The arrow indicates rearranged chromosomes.

## Data Availability

Not applicable.
